# Structure of Fungal α Mating Pheromone in Membrane Mimetics Suggests a Possible Role for Regulation at the Water-Membrane Interface

**DOI:** 10.3389/fmicb.2020.01090

**Published:** 2020-06-05

**Authors:** Angélica Partida-Hanon, Moisés Maestro-López, Stefania Vitale, David Turrà, Antonio Di Pietro, Álvaro Martínez-del-Pozo, Marta Bruix

**Affiliations:** ^1^Department of Biological Physical Chemistry, Institute of Physical Chemistry Rocasolano, CSIC, Madrid, Spain; ^2^Department of Biochemistry and Molecular Biology, Faculty of Chemistry, Complutense University, Madrid, Spain; ^3^Departmento de Genética, Universidad de Córdoba and Campus de Excelencia Agroalimentario (ceiA3), Córdoba, Spain

**Keywords:** peptide structure, membrane, disulfide bond, NMR, *Fusarium*, *Saccharomyces*

## Abstract

*Fusarium oxysporum* is a highly destructive plant pathogen and an emerging pathogen of humans. Like other ascomycete fungi, *F. oxysporum* secretes α-pheromone, a small peptide that functions both as a chemoattractant and as a quorum-sensing signal. Three of the ten amino acid residues of α-pheromone are tryptophan, an amino acid whose sidechain has high affinity for lipid bilayers, suggesting a possible interaction with biological membranes. Here we tested the effect of different lipid environments on α-pheromone structure and function. Using spectroscopic and calorimetric approaches, we show that this peptide interacts with negatively charged model phospholipid vesicles. Fluorescence emission spectroscopy and nuclear magnetic resonance (NMR) measurements revealed a key role of the positively charged groups and Trp residues. Furthermore, NMR-based calculation of the 3D structure in the presence of micelles, formed by lipid surfactants, suggests that α-pheromone can establish an intramolecular disulfide bond between the two cysteine residues during interaction with membranes, but not in the absence of lipid mimetics. Remarkably, this oxidized version of α-pheromone lacks biological activity as a chemoattractant and quorum-sensing molecule. These results suggest the presence of a previously unidentified redox regulated control of α-pheromone activity at the surface of the plasma membrane that could influence the interaction with its cognate G-protein coupled receptor.

## Introduction

**FIGURE 1:**
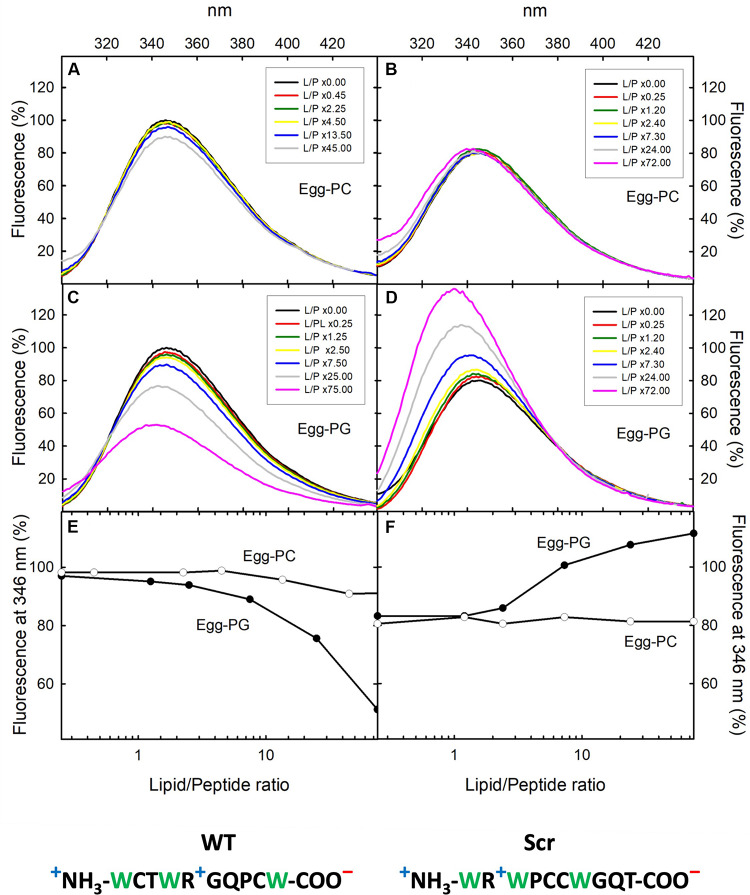
Fluorescence emission spectra. *F. oxysporum* α-pheromone peptides WT **(A,C)** and Scr **(B,D)** titrated with egg-PC or egg-PG MLVs. Peptide concentration was 8 μM in 50 mM sodium phosphate, pH 7.0. The phospholipid/peptide (L/P) molar ratios employed are indicated in the corresponding insets. Excitation wavelength was 275 nm. Fluorescence emission at 346 nm versus L/P molar ratios is shown for α-pheromone WT **(E)** and Scr versions **(F)**. Emission of the WT peptide at 346 nm in the absence of lipids was considered 100% for normalization of spectra. Sequences of WT (left) and Scr (right) peptides are indicated at the bottom, Trp residues are in green and formal charges under physiological conditions are indicated: “+”positive (blue) and “–“negative (red).

The ascomycete fungus *Fusarium oxysporum* is a highly destructive plant pathogen and an emerging pathogen of humans. Recently, mating pheromone α, a small peptide of 10 amino acids (^+^NH_3_-WCTWR^+^GQPCW-COO^–^, Figure 1) secreted by *F. oxysporum* was shown to function as a growth regulator, a chemoattractant and also as a quorum-sensing signal (Turrà and Di Pietro, 2015; Turrà et al., 2015; Vitale et al., 2019). Interaction of α-pheromone with the plasma membrane G-protein coupled receptor (GPCR) Ste2 (Turrà et al., 2015; Vitale et al., 2019) leads to the activation of a conserved mitogen-activated protein kinase (MAPK) pathway, which triggers chemotropic growth (Lee et al., 2010; Turrà et al., 2015) and regulates fungal community behavior via inhibition of spore germination (Vitale et al., 2019). In addition, α-pheromone also inhibits growth and cell division in a Ste2-independent manner. How α-pheromone-GPCR interaction induces these cellular responses is currently unknown. Nuclear magnetic resonance (NMR) determination of the high-resolution structure of *F. oxysporum* α-pheromone in H_2_O and trifluorethanol (TFE) revealed the presence of a key central β-turn resembling that of its yeast counterpart. Disruption of this fold by D-alanine substitution of the conserved central Gly6-Gln7 residues or by random sequence scrambling demonstrated a crucial role for this structural determinant in triggering receptor-dependent responses (Vitale et al., 2017). Thus, despite its short length, α-pheromone sequence contains enough information for non-trivial structure–function relationships and regulate these diverse biological processes. Interestingly, 3 of its 10 residues are Trp, an amino acid whose sidechain displays a high affinity for lipid bilayers (De Antonio et al., 2000; De Planque et al., 2002, 2003; Hong et al., 2013; García-Linares et al., 2016). Trp is abundant in membrane proteins and preferentially resides close to the lipid-water interface where it has a significant anchoring role (De Planque et al., 2003; De Jesus and Allen, 2013). A role of Trp in modulating responses to hydrophobic mismatch would explain how lipid composition could control the function of a range of membrane-active peptides and proteins. Because α-pheromone interacts with GPCRs, which are embedded in the plasma membrane, we asked whether lipidic environments affect its structural arrangement. Here we studied the interaction of α-pheromone with phospholipid bilayers (PC and PG vesicles) by following changes in peptide Trp fluorescence emission spectra and by using two different calorimetric approaches. We also compared the structures of wild-type α-pheromone (WT) and its non-functional derivative Scrambled (Scr), obtained by randomly changing the amino acid sequence, over long time periods in different micellar environments, using solution NMR. Our results provide new insights into the membrane interaction and redox-driven regulation of α-pheromone, as well as on its effect on biological activity in *F. oxysporum*, including both Ste2-dependent and independent processes.

## Materials and Methods

### Chemicals and Peptides

Egg-phosphatidylcholine (egg-PC), egg-phosphatidylglycerol (egg-PG), 1,2-dipalmitoyl-sn-glycero-3-phosphocholine (DPPC), and 1,2-dipalmitoyl-sn-glycero-3-phosphorylglycerol (DPPG) were obtained from Avanti Polar Lipids (Alabaster, AL, United States). Deuterated compounds (D_38_) DPC (dodecylphosphocholine) (98%), SDS (sodium dodecylsulfate) and D_2_O (99.9%) were from Cambridge Isotope Laboratories (United States). The percentages of deuteration are indicated in parenthesis. Non-deuterated DPC and SDS were from Avanti and Sigma, respectively. Sodium trimethylsilylpropanesulfonate (DSS) was from Sigma. Gemini surfactant [pentanediyl-1,5-bis(Hydroxyethylmethylhexadecylammonium bromide)] was synthesized by Dr. Razieh Amiri, University of Isphahan, Iran purified by three rounds of crystallization. Purity was >98%, as determined by TLC and NMR. The estimated CMC of this surfactant is about 2–3 μM at 25°C (Rosen and Tracy, 1998). Synthetic *F. oxysporum* WT α-pheromone (Trp-Cys-Thr-Trp-Arg-Gly-Gln-Pro-Cys-Trp; reduced and oxidized versions), a scrambled version (Scr) thereof (Trp-Arg-Trp-Pro-Cys-Cys-Trp-Gly-Gln-Thr) (Figure 1), and the dialanine substituted analogs D-Ala^1,2^ and D-Ala^6,7^ were obtained from GenScript (Piscataway, NJ, United States) or CASLO ApS (Denmark). All peptides were purified by HPLC to purities over 97.7% and lyophilized.

### Lipid Vesicles Preparation

Multilamellar (MLVs) and large unilamellar (LUVs) phospholipid vesicles were prepared as previously described (Martínez-Ruiz et al., 2001; García-Linares et al., 2013; Alm et al., 2015; Rivera-De-Torre et al., 2017). Briefly, a phospholipid solution in 2:1 (v/v) chloroform/methanol was first dried under a flow of nitrogen and then subjected to vacuum to remove residual solvents. The dry film obtained was used to prepare a lipid dispersion by adding 0.5–2.0 ml of 50 mM sodium phosphate buffer, pH 7.0, briefly vortex mixing, and incubating for 1 h at 37°C. When needed, this suspension of MLVs was further subjected to five cycles of extrusion at 37°C through polycarbonate filters (100-nm pore size) (Nucleopore, Whatman) to obtain a homogeneous population of LUVs. Laser scattering measurements were periodically conducted at the Spectroscopy and Correlation Facility of the Universidad Complutense to confirm LUV size homogeneity.

### Fluorescence Emission Characterization

Fluorescence emission spectra were conducted in a SLM Aminco 8000 spectrofluorimeter (Urbana, IL, United States), using 4 nm slits for both excitation and emission beams. The spectra were only recorded for excitation at 275 nm due to the absence of Tyr residues in the peptide sequence. Thermostated cells of 0.2 and 1.0 cm optical paths were used for the excitation and emission beams, respectively. Temperature was controlled with a circulating water bath (Huber Polystat) (De Antonio et al., 2000; Pardo-Cea et al., 2011; García-Linares et al., 2014). Samples were dissolved in 50 mM sodium phosphate buffer, pH 7.0, at the concentrations indicated and equilibrated at each temperature for at least 10 min prior to measurement.

### Isothermal Titration Calorimetry

The interaction between the peptides and LUVs was measured by isothermal titration calorimetry (ITC) as described before (Alegre-Cebollada et al., 2008; Maula et al., 2013; García-Linares et al., 2014; Rivera-De-Torre et al., 2017), using a VP-ITC calorimeter (Malvern MicroCal Worcestershire, United Kingdom). Briefly, 20.0–80.0 μM peptide solutions were titrated by injection of 20 μL aliquots of lipid suspensions (phospholipid concentration of 10.0 mM) at a constant temperature of 25°C. The buffer employed was 50 mM sodium phosphate, pH 7.0. Binding isotherms were adjusted using the standard MicroCal software with the OneSites model available within the Origin program where the “*n*” value refers to an average mean value of lipids affected by peptide binding to the bilayer. This does not necessarily imply specific direct contact between all n lipid molecules and the peptide. The *c* values (*c* = K_a_ × P_0_) for all the graphs were in the range 1–1000, the needed requirement in order to produce thermograms with the curvature required for the simultaneous determination of K_a_ and ΔH (Wiseman et al., 1989; Alegre-Cebollada et al., 2008). K_a_ is the affinity constant calculated according to the mentioned software model and P_0_ is the peptide concentration employed in the experiment.

### Differential Scanning Calorimetry

Differential scanning calorimetry (DSC) was performed essentially as previously reported (Gasset et al., 1995a, b; Cañadas et al., 2008) in a MicroCal VP differential scanning calorimeter (Microcal Inc., Northampton, MA, United States). The heating rate employed was 0.5°C/min. The experiments were conducted by loading DPPG or DPPC MLVs (1.0 mM), in the absence and presence of two different concentrations (24.0 or 95 μM) of WT α-pheromone, into the sample cell of the microcalorimeter. The corresponding buffer (50 mM sodium phosphate, pH 7.0) was placed in the reference cell. Twenty calorimetric scans were collected from each sample between 20 and 55°C. The standard MicroCal Origin software was used for data acquisition and analysis. The excess heat capacity functions were obtained after subtraction of the buffer baseline.

### Dynamic Light Scattering

Particle size and polydispersity index of aged samples of WT and Scr peptides (61 μM) in H_2_O, DPC or Gemini were determined by dynamic light scattering (DLS) with a DynaPro MS/X (Wyatt Inc.) spectrometer. Twenty acquisitions of 10 s at 25°C were obtained. Water was used as blank for data analysis; 30 mM DPC and a fresh WT α-pheromone solution were used as control.

### Nuclear Magnetic Resonance

Nuclear magnetic resonance (NMR) samples of WT and Scr peptides were prepared at 0.1–0.5 mM concentration in H_2_O/D_2_O (9:1 ratio by volume), DPC (20 mM), and SDS (20 mM) or Gemini (2 mM) at pH 5.0. All samples contain DSS as internal reference for ^1^H chemical shifts. The only deuterated lipid mimetic readily available for NMR and containing a negatively charged polar head, is SDS. Thus, membrane-like assays were performed with both fully or half-deuterated DPC and SDS. Gemini was only used in the protonated form. Spectra were recorded on a range of 5 and 40°C on a Bruker spectrometer equipped with a cryoprobe and operating at 800 MHz for the proton. Measurements were performed over time, spanning several weeks (from 0 to 260 days) depending on the media, in order to analyze sample evolution in H_2_O and micelles. The pH of all samples was checked and maintained constant over all the experiments. Between measurements, NMR samples were maintained at 25°C.

Phase-sensitive two-dimensional total correlated spectroscopy (TOCSY) and nuclear Overhauser enhancement spectroscopy (NOESY) spectra were recorded by standard techniques using the time-proportional phase increment mode. Water signal was suppressed by either presaturation or by using a 3-9-19 pulse sequence. TOCSY spectra were obtained by using 60 ms DIPSI2 with z filter spin-lock sequence. NOESY mixing time was 150 and 50 ms in micelle media. ^1^H-^13^C heteronuclear single quantum coherence (HSQC) spectra were recorded at ^13^C natural abundance. Number of scans were optimized depending of the experiment, typically 32–64 for ^1^H experiments and up to 256 for heteronuclear HSQC. Data were processed with the standard TOPSPIN program (Bruker Biospin, Karlsruhe, Germany). The 2D data matrices (2 K × 512 w) were multiplied by a square-sine-bell window function with the corresponding shift optimized for every spectrum and zero-filled (2 K × 1 K) prior to Fourier transformation. Baseline correction was applied in both dimensions. ^13^C δ-values were indirectly referenced by using the IUPAC-IUB recommended ^1^H/^13^C chemical shift ratio (Markley et al., 1998).

Assignments of the ^1^H spectra were done following the sequential assignment protocols (Wüthrich, 1986) with the help of the SPARKY software (Goddard and Kneller, 2005). The ^13^C resonances were identified based on the correlations between the protons and the bound carbon atoms present in the ^1^H-^13^C-HSQC spectra. Both peptides exhibited limited solubility in H_2_O and SDS and were generally more soluble in DPC and Gemini. Thus, in some cases, the evaluation of weak NOE signals and the complete assignment of the minority isomer (Xxx-Pro *cis*) in the equilibrium were difficult or impossible. NMR assignments are reported in Supplementary Tables S1–S7.

Structure calculations of the *trans* Xxx-Pro bond forms were done with CYANA 2.1 program (Günter, 2004). Small peptides do not adopt a unique, highly stable structure, but rather an ensemble of preferred, similar and modestly stable conformers, which are in equilibrium with low-populated conformers. To characterize the preferred conformers, medium- and long-range NOEs were selected. NOE integrated cross-peaks were translated into distance restraints, and the Φ and Ψ dihedral angle restraints were obtained using TALOS+ webserver (Shen et al., 2009). These angular restraints are in fully agreement with conformational chemical shifts. Typically, 200 structures were calculated using a standard protocol. The lists of distance constraints were checked with the corresponding NOESY spectra; ambiguous constraints were relaxed or removed in order to generate a final list used as input for a standard simulated annealing CYANA 2.1 calculation. The 20 conformers with the lowest target function values were selected and minimized. The structural ensembles were visualized and examined using MOLMOL (Koradi et al., 1996) and PyMOL (Schrödinger, 2010).

Micelle-peptide interactions were identified based on ^1^H assignment of both components and evaluated from intermolecular NOEs. For the analysis of the intermolecular contacts, the intensities of all NOEs between the micelle and the protons of each residue were added and scaled with respect to a known distance, and well-separated correlation signal, the intraresidual tryptophan H_δ 1_-H_ε 1_ NOE cross peak. To envisage the interaction, visual models were constructed using these NOEs in a qualitative way to accommodate a representative conformer of WT and the Scr peptides in a membrane representation.

### Fungal Strains, Quantification of Fungal Chemotropism and of Germ Tube Length

*Fusarium oxysporum* f. sp. *lycopersici* strain 4287 (race 2) and a previously generated *ste2*Δ mutant (Turrà et al., 2015) were cultured as previously described (Di Pietro et al., 2001). Microconidia production and storage was performed following previously reported procedures (Di Pietro et al., 2001).

To quantify hyphal redirectioning, freshly obtained microconidia were exposed to gradients of synthetic peptides by using a chemotropic plate assay (Turrà et al., 2015). All experiments were performed on at least five independent batches of cells (*n* = 100 cells per batch) per condition and repeated at least twice. Comparisons between the different tested conditions were performed by using a Yates’ corrected Chi-squared test (two-sided).

Germ tube length quantification was performed by applying 378 μM concentrations of the tested peptides, as previously described (Vitale et al., 2017). Fifty germ tubes per treatment were measured in at least four independent experiments. Statistical analysis was conducted using *t*-tests. All peptides were dissolved in 50% (v/v) methanol and assayed at the indicated concentrations. Visual inspection or image acquisition for directed growth and germ tube length assays was performed by the use of an Olympus BH2 binocular microscope (Olympus Iberia, Barcelona, Spain) (200× magnification) or a Zeiss Axio Imager M2 microscope (Zeiss, Barcelona, Spain; 400× magnification) using the following Zeiss filter block: DAPI (G 365, FT 395, LP 420) and the AxioVision 4.8 software (Zeiss), respectively. The length of individual germ tube was measured with the ImageJ software (Schneider et al., 2012).

### Quantification of Conidial Germination

To test the effect of pheromone peptides and variants thereof on *F. oxysporum* conidial germination, freshly obtained microconidia were incubated for 13 h at 28°C and 170 rpm in germination medium (GM) as previously described (Vitale et al., 2019). Synthetic *F. oxysporum* α-pheromone and its synthetic or oxidized variant versions (D-Ala^6,7^, D-Ala^1,2^, Cys^2^-Cys^9^), were added at 378 μM concentration to GM medium either alone or in combination with 0.3 mg ml^–1^ trypsin (Sigma-Aldrich). The percentage of germinated conidia was counted using differential interference contrast imaging on an Olympus BH2 microscope (400× magnification). All experiments were performed at least three times and the germination events from 300 or more conidia were counted for each experimental condition. Statistical differences between treatments were assessed by using a Yates’ corrected Chi-squared test (two-sided).

## Results

### α-Pheromone Interacts With Negatively Charged Phospholipid Vesicles

Wild-type α-pheromone and Scr peptides were titrated with egg-PC and egg-PG MLVs, and their interaction with the lipid vesicles was followed by registering Trp fluorescence emission spectra as a function of increasing phospholipid concentration (Figure 1). Only very small changes were detected for the egg-PC vesicles (Figures 1E,F), suggesting a lack of interaction. By contrast, both WT and Scr associated to egg-PG MLVs (Figure 1), most likely driven by electrostatic interactions resulting from their net positive charge of +1. For both peptides, the Trp fluorescence emission spectrum was centered at 346 nm in the absence of lipid vesicles, as expected from a Trp population exposed to the polar solvent (Figures 1C,D). Upon addition of egg-PG vesicles, the emission maximum for the WT and Scr peptides was blue-shifted to 342 and 340, respectively, suggesting a displacement of the indole side chains to a more hydrophobic microenvironment. In contrast to this similarity in the shift, the overall spectroscopic behavior of WT and Scr peptides in the presence of PG lipids differed dramatically. Whereas Scr displayed a significant quantum yield increment, attributable to hydrophobic indole shielding provided by the lipidic moiety (Figures 1D,F), in the WT peptide the blue shift was accompanied by a decrease of Trp fluorescence emission (Figures 1C,E). These findings suggest the establishment of local quenching interactions involving the Trp sidechains in the WT version of α-pheromone.

**FIGURE 2:**
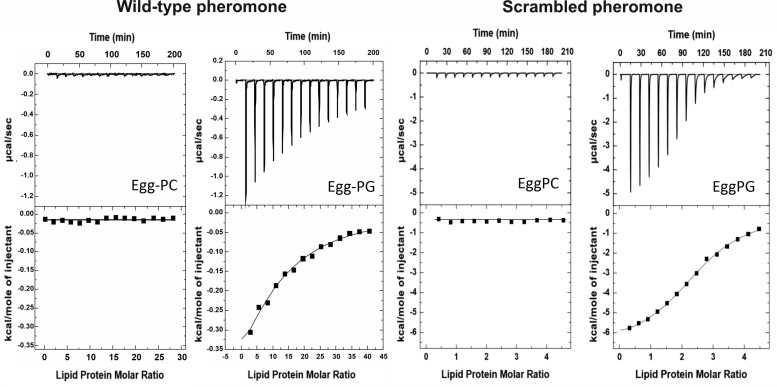
Binding of WT and Scr α-pheromone peptides to egg-PC or egg-PG MLVs measured by isothermal titration calorimetry (ITC). Peptide solutions at 20–80 microM were titrated by injection of 20 microL aliquots of lipid suspensions (10.0 mM). Experiments were conducted at 25°C.

Next, we employed ITC to quantify the interaction between peptide and lipid vesicles. Neither WT nor Scr produced a detectable heat signal when titrated with the egg-PC LUVs (Figure 2), in agreement with the fluorescence emission spectra shown in Figure 1. However, both peptides bound weakly to egg-PG vesicles (Figure 2). Binding was mostly enthalpy-driven, with an almost negligible entropic contribution in the case of the Scr peptide (Table 1). The K_a_ of the Scr peptide was about 24-fold higher than that of the WT pheromone (Table 1), when considering the affinity against individual phospholipid molecules. Thus, WT α-pheromone binding is weaker but affects a higher apparent number of phospholipid molecules (Table 1), confirming different modes of interaction of the two peptides.

**TABLE 1:** Binding of *F. oxysporum* WT α-pheromone and Scr peptides to egg-PG LUVs determined by ITC.

**Peptide**	**WT**	**Scr**
K_a_ (M^–1^)	1.2⋅10±32.6⋅102	2.8⋅10±41.8⋅103
*n*	10 ± 2.4	2.6 ± 0.1
ΔH (cal/mol)	-8⋅10±22.4⋅102	-6.7⋅10±31.0⋅102
ΔS (cal/mol⋅K)	11.3 ± 0.5	−2.1 ± 0.5

**n*, average mean value of lipids affected by peptide binding to the bilayer, which does not necessarily imply specific direct contact between all these n lipid molecules and the peptide. Ka, affinity constant (see section “Materials and Methods”). Standard deviations are indicated.*

**FIGURE 3:**
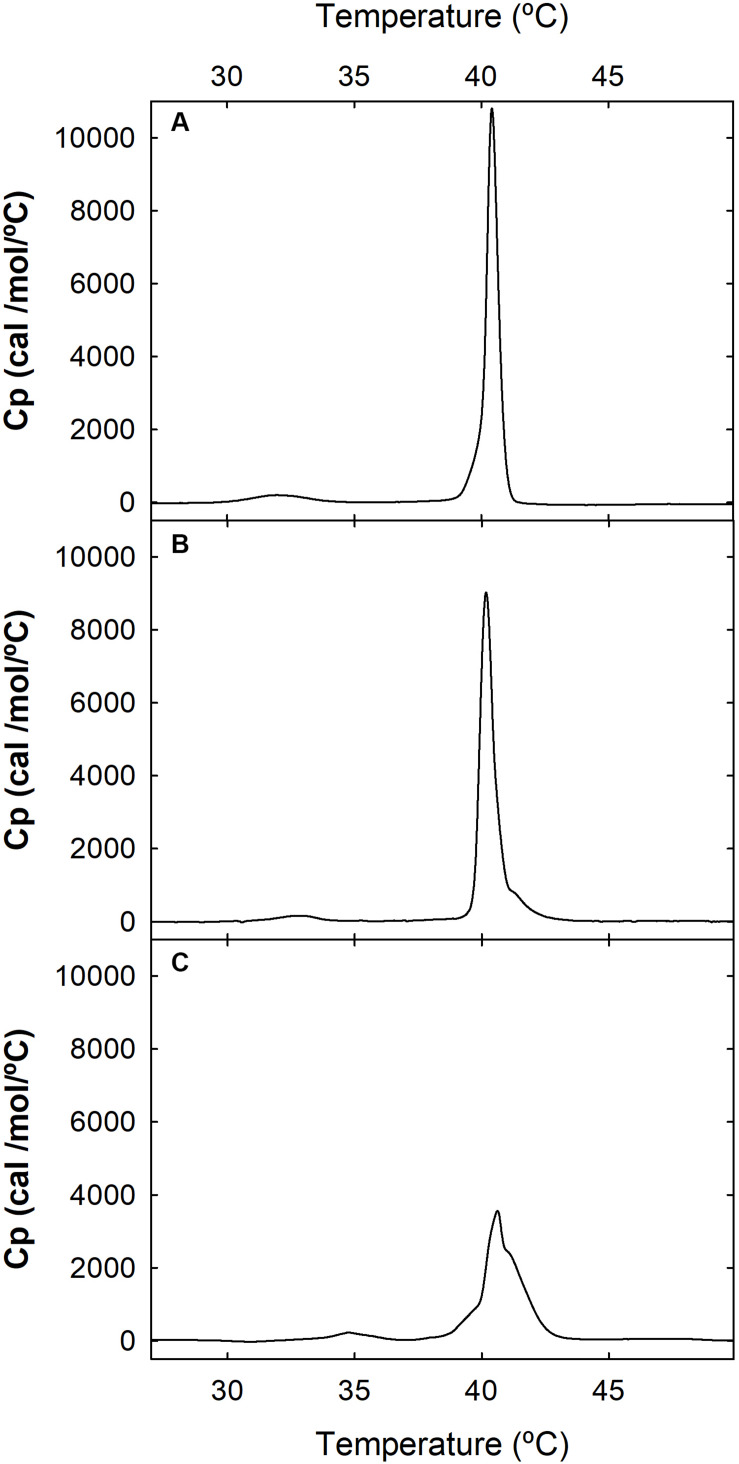
Differential scanning calorimetry (DSC). Thermograms of DPPG MLVs were recorded at a phospholipid concentration of 1.0 mM, in the absence **(A)** or presence of 24 μM **(B)** or 95 μM **(C)** WT α-pheromone. Buffer was 50 mM sodium phosphate, pH 7.0.

Based on the higher number of affected phospholipid molecules and the lower binding affinity to egg-PG LUVs of the WT α-pheromone, we hypothesized that it should be driven to the membrane by its Trp sidechains. This should be favored by electrostatic interactions between the peptide positive charges (Arg sidechain and amino N-terminal group). Once bound, it should remain anchored in a rather superficial arrangement, according to the role generally assigned to Trp side chains. Consequently, the interaction of WT α-pheromone with DPPG or DPPC MLVs was analyzed by DSC. In line with the prediction, the result of the WT peptide was consistent with a superficial interaction, as revealed by the displacement of discrete populations of phospholipid molecules to higher T_m_ values (Figure 3), as well as with a small change in the total amount of ΔH associated to the complete thermal transition (6900 cal/mol in the absence of peptide against 7100 and 5800 cal/mol for peptide concentrations of 24.0 and 95.0 μM, respectively). At the higher peptide concentration, we also observed a displacement of the pretransition to higher temperatures (Figure 3C). Overall, these results are consistent with the generation of superficially peptide-stabilized lipid domains, where both the electrostatic interactions and the Trp sidechains would play a key role in maintaining these within the membrane (Takahashi et al., 1992; Saenz et al., 2006). On the other hand, the corresponding DPPC thermograms failed to show a noticeable change at any of the peptide concentrations assayed with the DPPG vesicles (data not shown).

### Aging Yields Different Peptide Species Depending on the Environment

Currently, it is not feasible to use liquid NMR for studying peptide-lipid bilayer interactions in phospholipid vesicles, because the large size of the vesicles and the breadth of NMR signals prevents assignment. Therefore, information at atomic level resolution requires the use of lipid mimetics (Castrillo et al., 2010; García-Mayoral et al., 2010; Amiri et al., 2012a, b; Pulido et al., 2016). For this purpose, common surfactants such as DPC and SDS are widely used as membrane-like environments (Brown et al., 1981; Wider et al., 1982; Karslake et al., 1990; Castrillo et al., 2010). The micelles simulate the membrane interface due to the presence of a polar head group and a hydrophobic tail on each monomer, although perdeuterated samples are usually required. However, in some cases, high critical micelle concentration (CMC) values represent a disadvantage for their use in NMR because: (i) the monomers denature most globular proteins (Moosavi-Movahedi et al., 2005) and (ii) is difficult to determine whether the peptide is interacting with the micelle or with the individual monomers present in the equilibrium. By contrast, Gemini surfactants present low CMC values, smaller micelle sizes and a slow (millisecond) monomer-to-micelle-kinetics (Cui et al., 2008). Moreover, low Gemini concentrations are enough to trigger micelle formation (Amiri et al., 2012a, b), and no deuteration is required. Finally, the low percentage of monomers in the equilibrium ensures that peptide intermolecular interactions are triggered through the micelle structure. Collectively, these properties make Gemini surfactants excellent lipid-mimetics for NMR studies.

Starting from the known 3D structure adopted by α-pheromone in water solution (Vitale et al., 2017), we performed NMR analysis of WT and Scr in DPC, SDS and Gemini media. Although the solubility of the peptides in SDS was too low to study sample evolution or intermolecular interactions (see section “Materials and Methods”), it was sufficient to assign the corresponding ^1^H NMR signals. Different sets of signals were found in the NMR spectra of freshly dissolved samples, corresponding to those previously detected in water Xxx-Pro *trans/cis* bond conformational equilibrium (Vitale et al., 2017), confirming the *trans* conformer as the major species in all cases, except for Scr in Gemini media.

**FIGURE 4:**
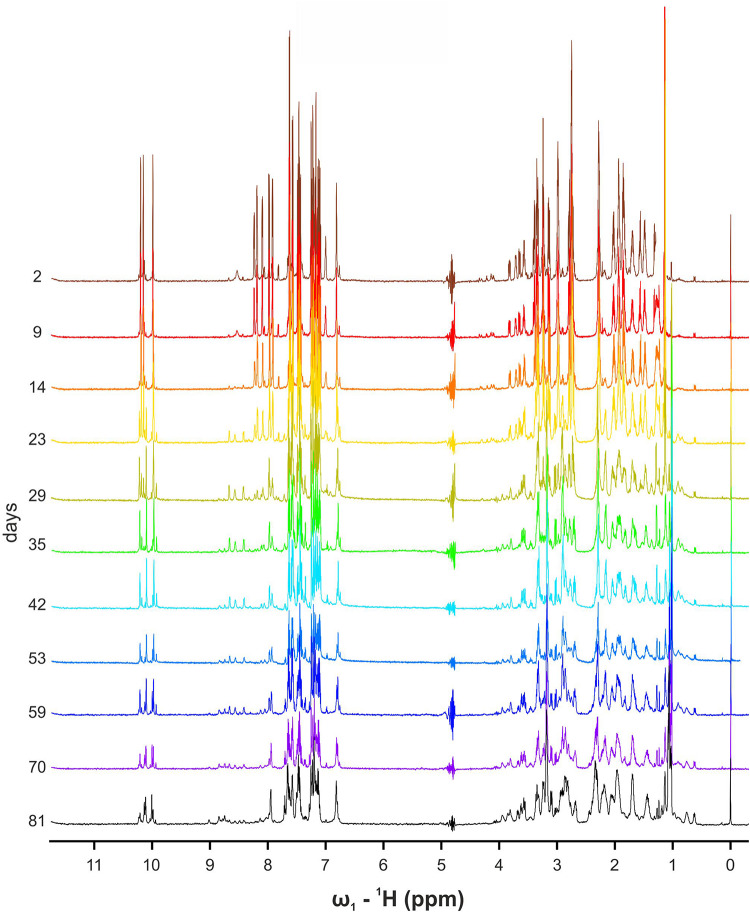
Overlapping 1D NMR spectra of WT α-pheromone. Spectra were obtained in H_2_O, pH 5.0, 25°C, as a function of time (days).

While performing these experiments, we noticed that the intensity of the original WT signals decreased and new ones appeared (Figure 4). Solubility of the peptide in water-solution decayed over time, and the overall intensities were significantly lower at the end of the experiment on day 81. These results suggested the formation of high molecular weight soluble aggregates. TOCSY, NOESY, and ^13^C-HSQC NMR spectra were recorded to obtain insights into the molecular entities present in the aged sample. TOCSY analysis, although not conclusive at the sequence-specific level, showed the presence of at least four different spin systems for each amino acid. Unfortunately, the complexity of the NOESY and HSQC spectra and the low concentration of the visible forms prevented an unambiguous assignment.

Dynamic light scattering analysis was also performed. The radius of the 30 mM DPC control (Supplementary Table S8) is in good agreement with values described in the literature (Kallick et al., 1995) and corresponds to a micelle containing approximately 40 DPC monomers. The monomeric form of WT α-pheromone in water yielded a single monodisperse peak corresponding to a radius of 2.3 nm, the expected size for the 3D peptide structure in solution (Vitale et al., 2017). By contrast, on day 102 approximately 71% of the particles in the aqueous solution were distributed according to an average radius larger than 196.6 nm (Supplementary Table S8), consistent with the formation of non-specific aggregates. The remaining 29% corresponded to monomodal monodisperse aggregates of around 38.6 nm. These aggregates containing a high number of monomers are most likely organized to optimize the burial of the Trp side chains and contain internal water molecules to solve the charges created by the N- and C-termini and the Arg side chains.

**FIGURE 5:**
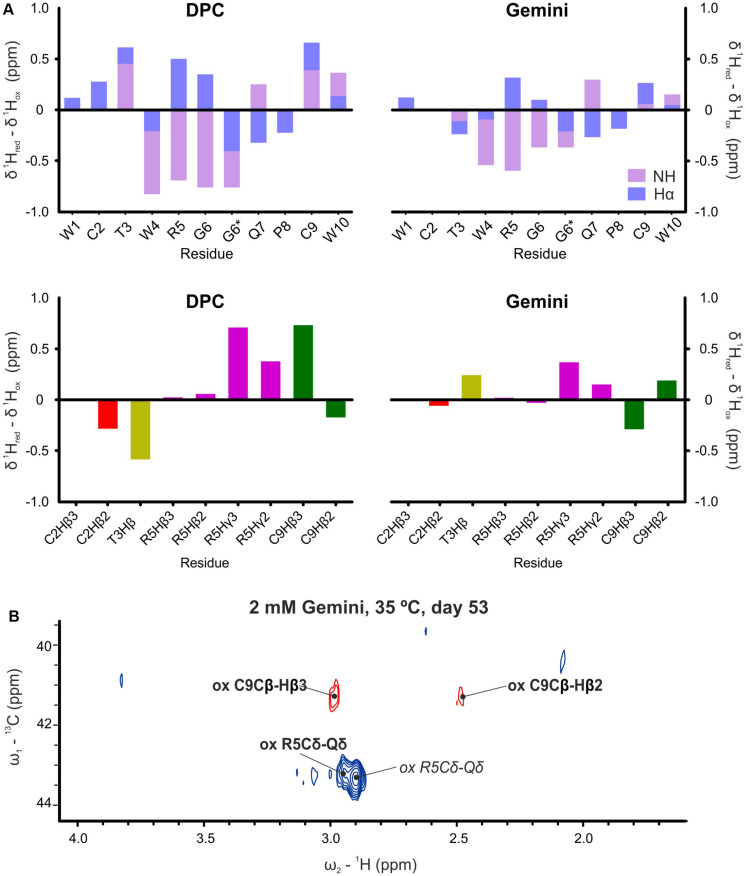
Proton chemical shift differences. **(A)** Backbone HN and Hα (top) and selected side chains (bottom) between reduced (day 0, fresh samples) and oxidized (more than 50 days, aged samples) forms of α-pheromone in DPC and Gemini. *Two lines are indicated for Gly6, since glycine has two Hα. **(B)** ^13^C-^1^H HSQC spectra of oxidized WT α-pheromone in Gemini micelles. Spectra were taken at 53 days after preparation. Signals corresponding to the *cis*-form are in italics.

In view of these results, peptide aging in the presence of micelles was also followed. While on day 0 the native reduced form of WT peptide was highly prevalent, we observed the progressive appearance of a monomeric oxidized form for both samples in DPC and Gemini micelles. Oxidation occurred between the two Cys residues of the peptide (Cys 2 and 9; Figure 1). The resulting intramolecular disulfide bond caused a dramatic change in the NMR parameters, including all backbone atoms and most side-chain protons (Figure 5A and Supplementary Tables S2, S5). Oxidation was explicitly revealed by changes in the chemical shift of the Cβ resonances, from 28 ppm in the reduced state (Vitale et al., 2017) to 41 ppm in the oxidized condition (Wishart et al., 1995; Figure 5A). In addition, the two Cys Hβ protons were clearly distinguishable in the oxidized version while only one single undistinguishable value was found for the reduced form. Regarding the remaining peptide signals, NH values of Trp4, Arg5, and Gly6 were also strongly affected by oxidation and displaced to larger chemical shift values (Figure 5B), whereas the Hα of Arg5 and Gly6 showed lower values. Altogether, these changes were consistent with an important conformational change of α-pheromone upon disulfide bond formation. Interestingly, in identical conditions, this progressive oxidation of the Scr peptide was not observed. Finally, DLS analysis in the presence of micelles revealed that both peptide (WT and Scr) preparations showed either a monodisperse or a polydisperse monomodal distribution consistent with radius values between 2.3 and 3.1 nm, which remained constant over time (Supplementary Table S8). Thus, it was concluded that in the presence of micelles α-pheromone did not aggregate.

### Three-Dimensional Structure of the Peptides in the Presence of Micelles

**FIGURE 6:**
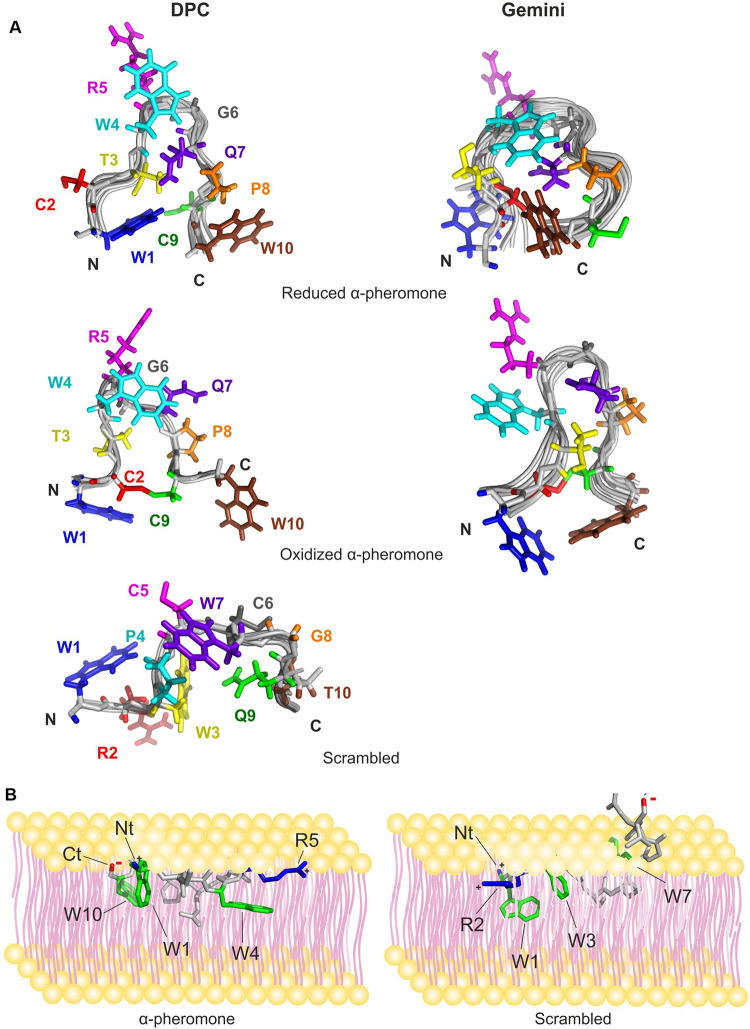
NMR solution structure of the peptides preferred conformations and a schematic representation suggesting their mode of interaction with the membrane surface. **(A)** The structures shown were calculated in DPC (left) and Gemini (right) for WT α-pheromone and Scr peptide. Superposition of the backbones of the best 20 structures in each family is shown in gray. Side chains of the energetically best structure in solution are color-marked depending on sequence position, 1, blue; 2, red; 3, yellow; 4, cyan; 5, magenta; 6, gray; 7, violet; 8, orange; 9, green; and 10, brown. N- and C-termini of the peptides are indicated by “N” and “C.” **(B)** *F. oxysporum* WT a-pheromone (left) and Scr peptide (right) are shown. The 3D structures of the peptides were obtained in this work by NMR. Residues important for the interaction are indicated; Trp rings are in green, positive charges in blue and negative charges in red. Peptide backbone is in gray.

The 3D structures of reduced and oxidized forms of WT and Scr peptides (*trans* Xxx-Pro form) in the presence of micelles were calculated based on the NMR data (Figure 6A and Table 2). The NOEs of reduced WT α-pheromone in DPC are compatible with a well-packed β-turn centered in Gly6 and Gln7 (Figure 6A), similar to that previously reported in a TFE-rich environment (Vitale et al., 2017). By contrast, the Scr peptide does not show a preferred secondary structure (Figure 6A) under these conditions. Both peptides displayed a higher degree of structural order in the presence of the surfactants as compared to water solution (Vitale et al., 2017). In terms of the different micelles employed, the reduced WT α-pheromone appeared to populate a more disordered ensemble in Gemini than in DPC. This result is likely due to the poorer quality of the NMR data, which is attributable to signal broadening resulting in reduced structural NOE information (Table 2). As expected, oxidized WT α-pheromone exhibited changes in the torsion of some backbone angles in both DPC and Gemini, due to the presence of the disulfide bond that alters the positions of the side chains (Figure 6A).

**TABLE 2:** Main structural statistical parameters for the ensemble of the 20 lowest target function conformers.

	**DPC**			**Gemini**	
	**WT (red)**	**WT (ox)**	**Scr (red)**	**WT (red)**	**WT (ox)**
Upper limit distance restraints (from NOEs)	167	77	95	117	84
φ/ψ Dihedral angle constrains (from chemical shifts)	12	8	11	9	12
Averaged CYANA target function value	0.52 ± 0.01	0.01 ± 0.01	0.18 ± 0.01	0.32 ± 0.03	0.68 ± 0.02
**Averaged maximum violation per structure:**					
Distance (Å)	0.03 ± 0.01	0.01 ± 0.06	0.008 ± 0.02	0.02 ± 0.03	0.006 ± 0.014
Dihedral angle (°)	1.06 ± 0.04	0.25 ± 0.01	1.28 ± 0.01	0.48 ± 0.33	1.76 ± 0.2
**Pairwise r.m.s.d. (Å):**					
Backbone atoms	0.30 ± 0.03	0.31 ± 0.20	0.39 ± 0.32	0.63 ± 0.24	0.40 ± 0.20
All heavy atoms	0.58 ± 0.22	1.16 ± 0.49	1.00 ± 0.34	1.92 ± 0.57	1.54 ± 0.58
**Ramachandran plot (%):**					
Favored	75.0	84.4	45.6	75.0	84.4
Additional	16.9	15.0	54.4	20.0	15.6
Outlier	8.1	0.6	0.0	5.0	0.0

*Data presented were calculated for WT and Scr α-pheromone in DPC and Gemini micelles, reduced (red) and oxidized (ox) forms.*

The Scr peptide maintained the preferred conformation for the *trans*-form in DPC, displaying a more compact and ordered conformation compared with the structure in water. In the presence of Gemini micelles, the preferred conformation was the *cis*-form although signal broadening made it impossible to calculate the 3D structure.

Compared to the reduced structures (Vitale et al., 2017), we noted that DPC and Gemini micelles favored the establishment of different Trp-Gln interactions in both peptides. In Scr, the Gln9 side chain tends to stay close in space respect to Trp3 (less than 5 Å), while in WT, Gln7 is close to Trp1, Trp4 and Trp10. In the latter case, very short distances (3.0–3.5 Å) were measured for some contacts between Trp-Gln side chains. If the structure adopted in micelles represents that in lipid vesicles, these short contacts could contribute to the significant decrease of Trp fluorescence emission observed in the WT peptide (Figure 1).

### Peptide Interaction With Micelles

**FIGURE 7:**
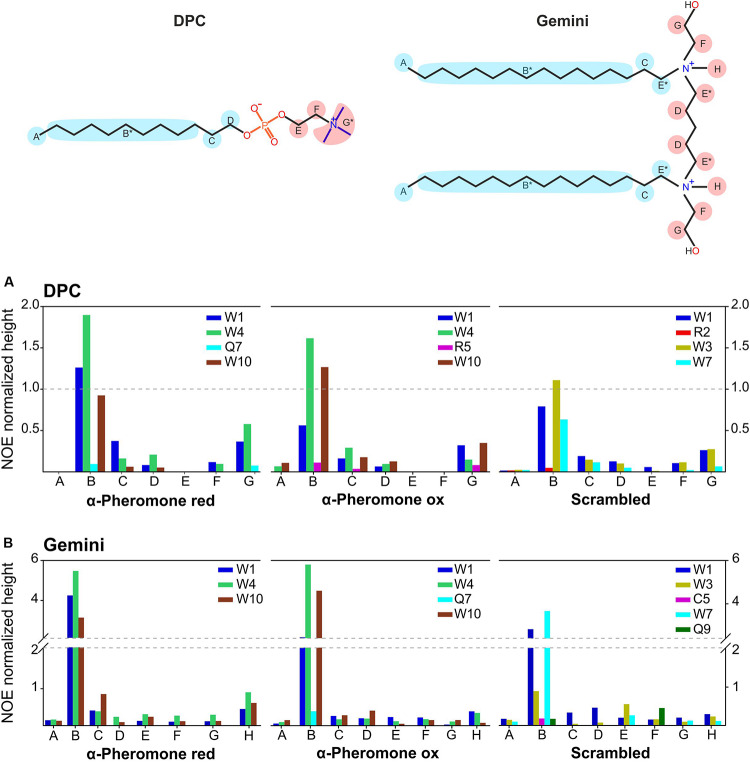
Scaled intermolecular NOEs. Bar plot, representing the scaled intermolecular NOEs for each residue found in reduced and oxidized WT α-pheromone and Scr peptides in DPC **(A)**, and in Gemini **(B)**. *Undistinguished protons appearing at the same chemical shift.

We used nuclear Overhauser effect, a powerful NMR tool to detect the close proximity of nuclei in the same molecule or between interacting partners. Qualitative evaluation of intermolecular NOE intensities revealed direct interactions between peptides and micelles (Figure 7). In the WT peptide, the Trp aromatic rings were crucial for the interaction with the hydrophobic moiety of DPC and Gemini micelles. However, the complex is dynamic since the contacts reach from the micelle core (B in DPC and Gemini) to the external protons (G in DPC and H in Gemini). These dynamics could explain the weak association observed with the PG vesicles (Figure 2). In all cases, the interaction strength based on NOE intensities from each group of protons was similar. As a rule, Trp10 appears to have a high relevance for the interaction when the WT peptide becomes oxidized. Apart from the aromatic groups, Gln7 and Arg5 are also contacting with the micelle. For Arg5, a snorkel model-type interaction common for Arg and Lys side chains (Strandberg and Killian, 2003) appears feasible. All these interactions are compatible with the superficial peptide-membrane interaction at the water-phospholipid heads interface, suggested by the DSC experiments (Figure 3). Similarly, the Scr peptide interacts preferentially with the hydrophobic region of DPC and Gemini micelles via the aromatic rings Trp1, Trp3, and Trp7. Differences in the contribution of these rings were observed depending on the medium, with Trp7 and Trp1 being more relevant in the interaction with Gemini.

As surfactants we employed lipid-mimetics rather than real membranes, since they represent the best tools available for peptide-lipid NMR measurements. Due to the intrinsically low sensitivity, the experiments were performed using very high peptide and lipid-mimetics concentrations. Therefore, our results provide a first proof of concept showing that α-pheromone peptide can interact with membranes by adopting a specific conformation and behavior. ITC and fluorescence characterization, performed at much lower concentrations and employing natural phospholipid mixtures (real bilayers), provide confirmation about the potential biological significance of the NMR observations. To test this biological significance of these findings, we next performed biological activity assays with the different peptide species.

### Cys Oxidation Affects Biological Activity of α-Pheromone

**FIGURE 8:**
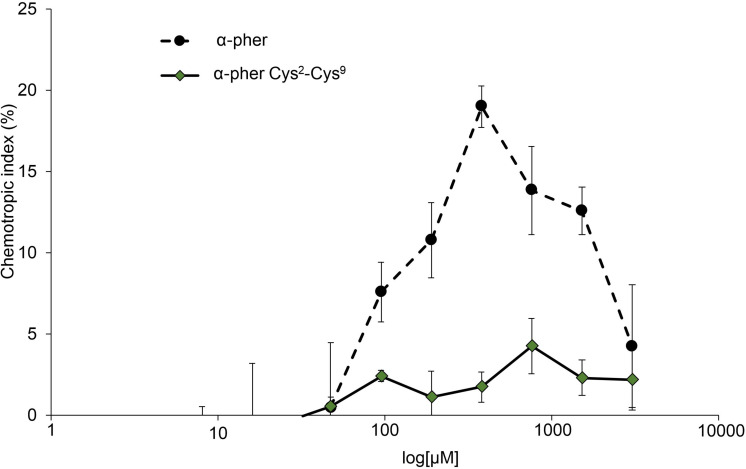
Dose-response curves for directed growth of *Fusarium oxysporum* germ tubes. Chemotropic growth of *Fusarium oxysporum* germ tubes was measured toward different concentrations of synthetic α-pheromone (α–pher) or its oxidized form (α–pher Cys^2^-Cys^9^). Data are presented as the mean from at least two independent experiments, each with five independent batches of cells (*n* = 100 germ tubes per batch). Error bars show SD.

**FIGURE 9:**
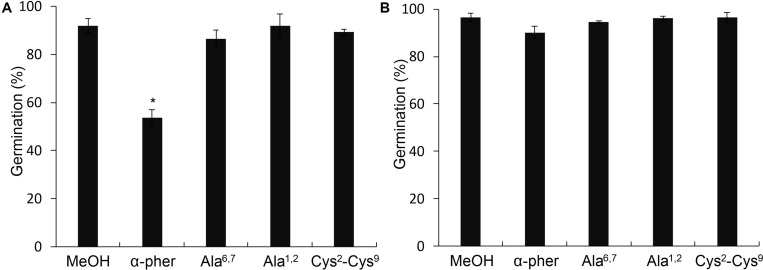
Germination of *Fusarium oxysporum* conidia. Germination at optimum concentration of inoculum (3.2 × 10^6^ conidia/ml) of the *F. oxysporum* WT **(A)** or *ste2*Δ **(B)** conidia in the presence of 378 μM α-pheromone (α-pher), its mutated (Ala^6,7^, Ala^1,2^) or oxidized (Cys^2^-Cys^9^) versions or the solvent methanol (*P < 0.0001 versus MeOH for a given pheromone treatment).

**FIGURE 10:**
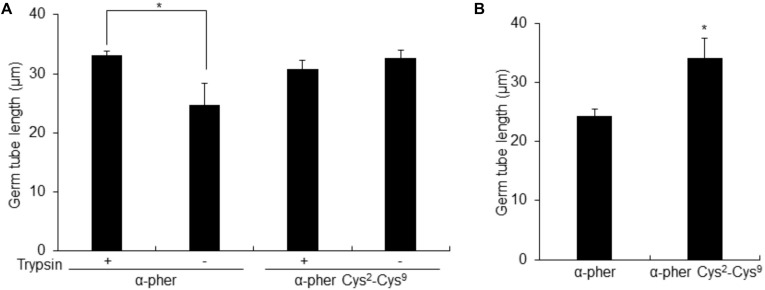
Length of *Fusarium oxysporum* conidial germ tubes. Length of *F. oxysporum* WT **(A)** or *ste2*Δ **(B)** germ tubes after 14 h exposure to a gradient of untreated (Trypsin –) or Trypsin-treated (Trypsin+) α-pheromone (α-pher) or its oxidized form (α–pher Cys^2^-Cys^9^) [*P < 0.02 versus trypsin-treated for a given pheromone treatment **(A)** or versus α-pheromone **(B)**]. Germ tube length was measured using the ImageJ software. Mean values were calculated from at least four independent experiments, each with 50 germ tubes. Error bars show SD.

Native reduced WT α-pheromone was previously shown to be perceived by *F. oxysporum* hyphae as a chemoattractant and a germination inhibitor in a Ste2-dependent manner (Turrà et al., 2015; Vitale et al., 2017, 2019). These studies also showed the lack of functionality of its Scr variant. Here we found that the Cys^2^-Cys^9^ oxidation version of α-pheromone abolishes its chemoattractant activity even when the oxidized peptide is tested at mM concentrations (Figure 8). In agreement with this, the quorum sensing activity of α-pheromone, which results in cell-density-dependent repression of conidial germination, was also abolished by Cys^2^-Cys^9^ oxidation or by D-Alanine substitution of the conserved central Gly6-Gln7 residues (Figure 9). Interestingly, a D-Ala^1,2^ analog which was previously shown to retain Ste2-dependent chemotropic activity (Vitale et al., 2017) also failed to repress conidial germination (Figure 9). Furthermore, α-pheromone growth inhibitory activity, which is independent of Ste2, was similarly reduced by trypsin-treatment (negative control) or Cys oxidation (Figure 10).

## Discussion

*Fusarium oxysporum*α-pheromone is a small secreted peptide with a unique sequence, which imparts specific developmental functions by interacting with the integral membrane protein Ste2 (Turrà et al., 2015; Vitale et al., 2017, 2019). We reasoned that in such a context, interaction of α-pheromone with the plasma membrane could be relevant for its structure and biological function. Here we investigated the interaction of α-pheromone with lipid membranes, using as a negative control Scr, a biologically inactive variant with a randomly scrambled sequence but identical amino acid composition. We found that both peptides interact with negatively charged PG but not with PC vesicles, suggesting (1) the presence of electrostatic interactions and (2) a key role for positively charged peptide groups such as the amino-terminal and sidechain guanidinium of the single Arg residue. Unexpectedly, binding of Scr to PC was stronger than that of WT α-pheromone. This is likely due to differences in the distribution of charged and hydrophobic groups between the two peptides, with WT showing a rather homogeneous distribution whereas Scr accumulates not only the more positive charges at the N-terminus but also its Trp residues appear clustering at a specific region. Together with the 3D structures in the presence of membrane-mimicking environments, this result suggests that the two peptides adopt different arrangements when interacting with phospholipid vesicles.

The high proportion of Trp (3 of 10 residues) and the fluorescence emission results (Figure 1), suggest a key role for this amino acid in membrane interaction. This idea was confirmed by NMR analysis of the interaction with micelles of the two surfactants DPC and Gemini. Contacts were more specific for WT α-pheromone than for Scr, most likely due to the differences in the positions of key amino acids such as Trp, Arg, and Gln. The finding that Scr has less contacts than WT with both types of micelles agrees with the lower number of phospholipid molecules affected by its interaction. Interestingly, we noted that NOE signals were representative of efficient interaction with Gemini micelles but not with the individual detergent monomers. Due to the low CMC value of Gemini, the equilibrium is fully shifted toward the formation of micelles rather than isolated surfactant molecules. The NMR parameters suggest that the interaction with micelles is highly dynamic, in agreement with the weak affinity constant of WT α-pheromone. Since both peptides, WT and functionally inactive Scr, interact very differently with lipids and lipid mimetics, those changes detected between both modes of interaction suggest that the WT peptide might be establishing biologically significant interactions.

The presence of two free sulfhydryl groups, Cys2 and Cys9, in α-pheromone suggested a possible role of redox processes in modulation of its structure. This idea was reinforced by the observation that α-pheromone underwent long-term aggregation in water solution. Unfortunately, NMR signal intensities were too low, and the spectra too complex, to unequivocally assign the changes detected. The most feasible explanation to this observation would be, however, the formation of disulfide bonds involving Cys2 and Cys9 sidechains, resulting then in massive oligomerization of the peptide. Disulfide bonds were also generated in the presence of micelles, albeit at a much lower rate, but these were determined to be intramolecular, specifically established between Cys2 and Cys9, and oligomerization of α-pheromone was not detected. This suggests that the micellar structure would protect the monomer scaffold, which most likely represents a biologically functional form of α-pheromone, but not necessarily its active conformation. The protective effect had to be caused by a conformational change in the peptide during interaction with the micelle, whereby the two SH groups which are distant in the free state, were accommodated in the correct arrangement for intra-molecular redox reactions. This will result in a more compact structure compared to that adopted in water, as expected from previous results obtained in the presence of a high proportion of TFE (Vitale et al., 2017). Similar effects have been also previously described for other peptidic hormones such as enkephalins, which are believed to interact with the membrane to adopt a conformation suitable for, in this case, their binding to its cognate receptor (Marcotte et al., 2004). Supporting the significance of these results, the Scr peptide, which carries two consecutive Cys residues, failed to undergo intramolecular oxidation or to form aggregates regardless of the presence of surfactant micelles. Overall, this set of results suggests the potential existence of an *in vivo* redox based mechanism of regulation, most probably with the intervention of non-yet detected specific enzymes.

Based on the calculated structures obtained in this work, we propose a mode of interaction of α-pheromone with membranes, represented as a cartoon in Figure 6B. According to this proposal, α-pheromone would position parallel or slightly tilted on the membrane surface, allowing the interaction of Trp 1 and Trp 10 rings (N- and C-peptide terms) with the hydrophobic regions of the lipids. Positive charges at the N-terminus and in the center of the β-turn (Arg 5) could help to anchor the structure at both ends to the phosphate groups. This model is in line with our observation, that the *in vitro* interaction of α-pheromone with lipids is dynamic, weak and superficial, and that the distribution of charge and hydrophobicity is a key factor for the interaction. The model also explains the higher number of lipid molecules affected by the interaction of WT pheromone compared to Scr, as it predicts the entire WT peptide to interact with the membrane, compared to only the N-terminus of Scr due to local accumulation of Trp residues with positive charge (Figure 6B).

After initial contact, the interaction of α-pheromone with the membrane would be further strengthened by the establishment of specific links that contribute to optimum orientation of the molecule and promote the conformational changes that lead to its oxidation. As suggested above, currently unknown redox-regulated enzymes could be responsible for *in vivo* oxidation of α-pheromone, since the herein observed non-enzymatic *in vitro* oxidation is too slow to be of real biological significance without the intervention of rate accelerating enzymes. According to this suggestion, we speculate that this interaction with the membrane could contribute to redox-mediated modulation; with low concentrations of α-pheromone favoring selective binding to its cognate receptor rather than low affinity binding to the lipid bilayer (see Figure 2). Another putative role of the membrane would be to sequester α-pheromone molecules to the cell surface. This would increase the efficiency of α-pheromone binding to the cognate GPCR Ste2, by increasing local peptide concentration while preventing the formation of peptide aggregates.

## Conclusion

Consequently, our discovery of an oxidized version of α-pheromone immediately suggested a mechanism of redox-mediated control of its biological function. In support of this idea, we found that all the known *in vivo* effects of α-pheromone were abolished upon the establishment of the disulfide bond between Cys2 and Cys9. Loss of biological activity of oxidized pheromone was comparable to that previously reported for other biologically inactive versions such as Scr or the Ala^1,2^ and Ala^6,7^ substituted versions, and mimicked the effect of peptide degradation with trypsin (Turrà et al., 2015; Vitale et al., 2017, 2019). Collectively, these results reinforce the hypothesis of a new role for the cellular redox environment in regulating biological activity α-pheromone, a fascinating class of small signaling molecules. Intriguingly α-pheromone from the budding yeast *Saccharomyces cerevisiae* does not contain Cys residues in its 13-residue sequence (Kurjan and Herskowitz, 1982), suggesting that redox control of α-pheromone activity might have specifically evolved in certain ascomycete fungi.

## Data Availability Statement

The datasets generated for this study are available on request to the corresponding author.

## Author Contributions

AP-H and MB designed, did and interpreted the NMR experiments. MM-L and ÁM designed, did and interpreted the spectroscopic and calorimetric characterization. SV, DT, and AD performed the *in vivo* functional characterization. All authors participated in writing the manuscript as well as in making corrections and suggestions and taking active part of discussions and interpretations.

## Conflict of Interest

The authors declare that the research was conducted in the absence of any commercial or financial relationships that could be construed as a potential conflict of interest.

## Abbreviations

CFW: calcofluor white
CMC: critical micellar concentration
DLS: dynamic light scattering
DPC: dodecylphosphocholine
DPPC: 1,2-dipalmitoyl-sn-glycero-3-phosphocholine
DPPG: 1,2-dipalmitoyl-sn-glycero-3-phosphorylglycerol
DSC: differential scanning calorimetry
DSS: sodium trimethylsilylpropanesulfonate
GM: germination medium
GPCR: G protein-coupled receptor
HSQC: heteronuclear single quantum coherence
ITC: isothermal titration calorimetry
LUV: large unilamellar vesicle
MAPK: mitogen-activated protein kinase
MeOH: methanol
MLV: multilamellar vesicle
NMR: nuclear magnetic resonance
NOESY: nuclear Overhauser enhancement spectroscopy
PC: phosphatidylcholine
PDB: potato dextrose broth
PG: phosphatidylglycerol
Scr: scrambled
TOCSY: phase-sensitive two-dimensional total correlated spectroscopy
WA: water agarose
WT: wild-type.
