# Supplementary material for: Structure of Fungal α Mating Pheromone in Membrane Mimetics Suggests a Possible Role for Regulation at the Water-Membrane Interface
Source: Front Microbiol. 2020 Jun 5;11:1090. doi: 10.3389/fmicb.2020.01090 (PMC7289986; doi:10.3389/fmicb.2020.01090)
Supplement: Supplementary file 1 [file Data_Sheet_1.PDF]

Supplementary Material

Supplementary Tables

Table S1. ^{1}H and $^{13}C\alpha$ chemical shifts (ppm from DSS) of reduced α-pheromone Q-P *trans* conformation in 20 mM DPC, pH 5.0, 25ºC.

Table S2. ^{1}H and $^{13}C\alpha$ chemical shifts (ppm from DSS) of oxidized α-pheromone Q-P *trans* conformation in 20 mM DPC, pH 5.0, 25ºC.

Table S3. ^{1}H and $^{13}C\alpha$ chemical shifts (ppm from DSS) of reduced scrambled W-P *trans* conformation in 20 mM DPC, pH 5.0, 25ºC

Table S4. ^{1}H and $^{13}C\alpha$ chemical shifts (ppm from DSS) of reduced α-pheromone Q-P *trans* conformation in 2 mM Gemini, pH 5.0, 25ºC

Table S5. ^{1}H and $^{13}C\alpha$ chemical shifts (ppm from DSS) of oxidized α-pheromone Q-P *trans* conformation in 2 mM Gemini, pH 5.0, 25ºC

Table S6. ^{1}H and $^{13}C\alpha$ chemical shifts (ppm from DSS) of reduced scrambled W-P *cis* conformation in 2 mM Gemini, pH 5.0, 25ºC

Table S7. ^{1}H chemical shifts (ppm from DSS) of reduced α-pheromone W-P *trans* conformation in 30 mM SDS, pH 5.0, 25ºC.

Table S8. Dynamic Light Scattering analysis of α-pheromone and scrambled sequence in H_2O, DPC and Gemini, pH 5.0 at 25 ºC.

Table S1. ^{1}H and $^{13}C\alpha$ chemical shifts (ppm from DSS) of reduced α-pheromone Q-P *trans* conformation in 20 mM DPC, pH 5.0, 25ºC

Residue	HN	Hα	Hβ	Cα	Others H	
W1	----	4.32	3.42	53.85	HD1	7.39
					HE1	10.64
					HE3	7.62
					HH2	7.03
					HZ2	7.45
					HZ3	7.10
C2	8.60	4.42	2.70	55.86		
T3	8.25	4.38	4.10	59.10	QG2	1.12
W4	8.18	4.69	3.23	54.63	HD1	7.26
			3.26		HE1	10.56
					HE3	7.57
					HH2	7.02
					HZ2	7.43
					HZ3	7.08
R5	8.25	4.11	1.56	53.38	HE	7.30
			1.76		HG2	1.24
					HG3	1.31
					QD	3.02
G6	7.79	3.88		42.25		
		3.67				
Q7	7.88	4.54	1.89	55.51	HE21	6.82
			2.06		HE22	7.51
					QG	2.29
P8	----	4.32	1.68	60.42	HD2	3.60
			1.99		HD3	3.73
					HG2	1.87
					HG3	1.95
C9	8.14	4.42	2.81	55.68		
W10	7.60	4.52	3.19	55.53	HD1	7.22
			3.33		HE1	10.46
					HE3	7.56
					HH2	6.98
					HZ2	7.42
					HZ3	7.05

Table S2. [1]H and [13]Cα chemical shifts (ppm from DSS) of oxidized α-pheromone Q-P *trans* conformation in 20 mM DPC, pH 5.0, 25ºC

Residue	HN	Hα	Hβ	Cα	Others H	
W1	----	4.20	3.46	54.63	HD1	7.47
			3.50		HE1	10.87
					HE3	7.52
					HH2	7.33
					HZ2	7.53
					HZ3	7.14
C2	n.a.	4.14	2.70	n.a.		
			2.99			
T3	7.79	4.69	3.76	68.83	QG2	0.96
W4	9.00	n.a.	3.01	n.a.	HD1	7.14
			3.15		HE1	10.51
					HE3	7.70
					HH2	7.08
					HZ2	7.43
					HZ3	7.04
R5	8.94	3.60	1.53	53.95	HD2	2.80
			1.70		HE	7.23
					HG2	0.53
					HG3	0.88
G6	8.55	3.53		n.a.		
		4.07				
Q7	7.63	4.88	1.91	53.84	HE21	6.87
			2.16		HE22	7.53
					HG2	2.30
					HG3	2.38
P8	----	4.54	1.87	60.26	HD2	3.62
			2.31		HD3	3.79
					HG2	1.98
					HG3	2.07
C9	7.75	3.77	2.07	n.a.		
			2.98			
W10	7.24	4.38	3.15	55.41	HD1	7.15
			3.39		HE1	10.44
					HE3	7.41
					HZ2	7.32
					HH2	7.00
					HZ3	6.92

Table S3. [1]H and [13]Cα chemical shifts (ppm from DSS) of reduced scrambled W-P *trans* conformation in 20 mM DPC, pH 5.0, 25ºC

Residue	HN	Hα	Hβ	Cα	Others H	
W1	----	4.30	3.26	55.52	HD1	7.21
			3.35		HE1	10.17
					HE3	7.32
					HH2	6.47
					HZ2	7.24
					HZ3	6.73
R2	8.35	4.42	1.67	55.76	HE	7.28
			1.79		HG2	1.52
					HG3	1.55
					QD	3.14
W3	8.36	4.41	3.14	55.77	HD1	7.42
			3.36		HE1	10.74
					HE3	7.65
					HH2	7.08
					HZ2	7.50
					HZ3	7.12
P4	----	4.40	1.90	63.11	HD2	3.62
			2.18		HD3	3.97
					QG	1.90
C5	8.40	4.42	2.90	58.52		
C6	8.28	4.47	2.89	58.53		
W7	8.14	4.71	3.21	57.45	HD1	7.20
			3.33		HE1	10.44
					HE3	7.47
					HH2	6.94
					HZ2	7.38
					HZ3	7.02
G8	8.46	3.91		45.31		
		3.98				
Q9	8.19	4.41	2.01	54.44	HE21	6.85
			2.17		HE22	7.64
					QG	2.36
T10	7.88	4.16	4.24	63.15	QG2	1.17

Table S4. ^{1}H and $^{13}C\alpha$ chemical shifts (ppm from DSS) of reduced α-pheromone Q-P *trans* conformation in 2 mM Gemini, pH 5.0, 25ºC

Residue	HN	Hα	Hβ	Cα	Others H	
W1	----	4.26	3.30	56.18	HD1	7.28
			3.36		HE1	10.29
					HE3	7.58
					HH2	7.09
					HZ2	7.46
					HZ3	7.16
C2	8.38	4.49	2.70	n.a.		
T3	8.20	4.31	4.13	n.a.	QG2	1.12
W4	8.21	4.67	3.21	57.06	HD1	7.22
			3.24		HE1	10.23
					HE3	7.56
					HH2	7.08
					HZ2	7.44
					HZ3	7.19
R5	8.15	4.12	1.52	56.22	QD	3.01
			1.73		QG	1.29
G6	7.74	3.66		45.22		
		3.86				
Q7	7.92	4.50	1.84	n.a.	HE21	6.80
			2.01		HE22	7.45
					QG	2.27
P8	----	4.24	1.60	63.14	HD2	3.57
			1.86		HD3	3.71
					HG2	1.92
					HG3	1.95
C9	8.01	4.39	2.74	56.11		
W10	7.60	4.48	3.14	58.94	HD1	7.19
			3.31		HE1	10.11
					HE3	7.59
					HH2	7.04
					HZ2	7.42
					HZ3	7.12

n.a. not assigned

Table S5. [1]H and [13]Cα chemical shifts (ppm from DSS) of oxidized α-pheromone Q-P *trans* conformation in 2 mM Gemini, pH 5.0, 25ºC

Residue	HN	Hα	Hβ	Cα	Others H	
W1	----	4.16	3.31	56.37	HD1	7.32
					HE1	10.35
					HE3	7.58
					HH2	7.11
					HZ2	7.51
					HZ3	7.20
C2	n.a.	4.52	2.76	56.79		
T3	8.26	4.56	3.92	61.60	QG2	1.01
W4	8.69	4.78	3.16	57.01	HD1	7.21
			3.18		HE1	10.21
					HE3	7.64
					HH2	7.10
					HZ2	7.47
					HZ3	7.18
R5	8.69	3.83	1.50	56.59	HE	6.95
			1.76		HG2	0.92
					HG3	1.14
					QD	2.92
G6	8.06	3.58		45.37		
		4.07				
Q7	7.59	4.78	1.93	52.87	HE21	6.77
			2.16		HE22	7.43
					QG	2.32
P8	----	4.43	1.75	63.14	HD2	3.62
			2.21		HD3	3.81
					HG2	1.97
					HG3	2.06
C9	7.88	4.15	2.55	56.65		
			3.03			
W10	7.41	4.46	3.17	58.38	HD1	7.19
			3.33		HE1	10.09
					HE3	7.56
					HH2	7.03
					HZ2	7.40
					HZ3	7.07

n.a. not assigned

Table S6. ^{1}H and $^{13}C\alpha$ chemical shifts (ppm from DSS) of reduced scrambled W-P *cis* conformation in 2 mM Gemini, pH 5.0, 25ºC

Residue	HN	Hα	Hβ	Cα	Others H	
W1	----	4.40	n.a.	n.a.	HD1	7.25
					HE1	10.21
					HZ2	7.46
R2	n.a.	4.52	1.70	55.70	QD	3.09
			1.79		QG	1.54
W3	8.36	4.39	3.15	n.a.	HD1	7.30
			3.20		HE1	10.32
					HE3	7.58
					HZ2	7.50
P4	----	4.23			HD2	3.44
					QG	1.66
C5	8.19	4.34	2.76	56.21		
C6	8.08	4.56	2.72	55.86		
W7	8.02	4.40	3.15	n.a.	HD1	7.21
			3.25		HE1	10.16
					HE3	7.55
					HZ2	7.44
G8	8.12	3.77		n.a.		
		3.86				
Q9	8.08	4.37	1.96	55.85	QG	2.32
			2.12			
T10	7.84	4.15	4.22	63.13	QG2	1.14

n.a. not assigned

Table S7. [1]H chemical shifts (ppm from DSS) of reduced α-pheromone Q-P *trans* conformation in 30 mM SDS, pH 5.0, 25ºC.

Residue	HN	Hα	Hβ	Others H
W1	----	4.44	3.40	HD1 7.32
			3.45	HE1 9.94
				HZ2 7.41
C2	8.07	4.36	2.71	
T3	7.96	4.31	4.21	QG2 1.18
W4	7.83	4.64	3.25	HD1 7.26
			3.31	HE1 9.86
				HZ2 7.39
R5	7.69	4.09	1.41	QG 1.23
			1.57	QD 2.97
				QE 6.94
G6	7.59	3.84		
Q7	7.78	4.51	1.88	QG 2.27
			2.03	QE 6.71
P8	----	4.26	1.78	QG 1.81
			1.85	QD 3.54
C9	7.86	4.44	2.82	
W10	7.52	4.69	3.25	HD1 7.20
			3.32	HE1 9.84
				HZ2 7.39

Table S8. Dynamic Light Scattering analysis of α-pheromone (fresh and aged sample) and scrambled sequence in H_2O, DPC and Gemini pH 5.0 and 25 ºC, at different days from sample preparation (days in brackets), and same NMR conditions.

Molecule	R (nm)	Pd (%)	Mass (%)
DPC 30 mM	2.4	11.7	100
α-pheromone in H_2O (0 d)	2.3	0	99.5
α-pheromone in H_2O (102 d)	> 196.6	> 16.8	70.6
	38.6	0	29.4
α-pheromone in DPC (249 d)	2.3	30.1	100
α-pheromone in Gemini (305 d)	3.1	13.6	100
scrambled in H_2O (0 d)	14.2	0	100
scrambled in H_2O (674 d)	6.5	0	100
scrambled in DPC (449 d)	2.4	17.6	100
scrambled in Gemini (305 d)	2.7	15.2	99.8

R: Radius

Pd: Polydispersity
